# Beyond black and white: A framework for identifying grey literature in palliative care research

**DOI:** 10.1177/02692163251381487

**Published:** 2025-10-23

**Authors:** Raechel Damarell, Seth Nicholls, Jessica Tyndall, Caroline Phelan

**Affiliations:** 1 Research Centre for Palliative Care, Death and Dying (RePaDD), College of Nursing and Health Sciences, Flinders University, Adelaide, SA, Australia; 2 Torrens University, Adelaide, SA, Australia

**Keywords:** grey literature, systematic reviews as topic, information storage and retrieval, palliative care

## Abstract

**Background:**

Research evidence is fundamental to informing clinical decision-making and advancing palliative care practice. Although academic, peer-reviewed journals underpin evidence-based healthcare, they represent only part of the knowledge landscape. Incorporating grey literature from sources outside traditional academic publishing can: provide context, balance and diverse perspectives; address knowledge gaps; and mitigate publication bias. However, its decentralised and dispersed nature can pose challenges for researchers unfamiliar with its scope and diversity.

**Aim:**

To present a flexible framework comprising 12 elements to support researchers in systematically identifying and locating grey literature relevant to palliative care across a broad range of sources. The framework accommodates variation in research focus, available resources, and context. Practical guidance is also provided for reporting grey literature searches with the transparency required in systematic reviews.

**Methods:**

The framework was developed through expert consensus, informed by the authors’ collective experience in systematic review methodology, grey literature searching, and information retrieval. It has been iteratively refined through teaching and real-world review projects. Each included source was assessed for its depth and breadth of palliative care content.

**Results:**

The 12-element framework supports palliative care researchers in planning and executing searches across a wide range of fit-for-purpose sources. Practical examples are provided alongside a classification of grey literature source types.

**Discussion and conclusion:**

This framework offers structured yet adaptable guidance to support more consistent grey literature engagement. Persistent challenges include defining search boundaries, managing duplication, record-keeping, and assessing quality. Future research should explore the framework’s utility across diverse review types and palliative care research priorities.

**What is already known about the topic?**

- Grey literature offers valuable insights for palliative care but is challenging to locate due to its decentralised nature.
- It helps address publication bias and brings in patient- and carer-centred perspectives.
- Despite its value, there is no standard methodology for identifying and retrieving grey literature in palliative care research.

**What this paper adds?**

- A 12-element framework was developed to support flexible yet systematic searching of grey literature in palliative care research.
- This framework may improve access to non-traditional sources, supporting evidence-informed research and practice.
- Social media and generative artificial intelligence (AI) tools are emerging adjuncts for discovering grey literature.

**Implications for practice, theory, or policy**

- Grey literature can strengthen multidisciplinary, patient-centred approaches in palliative care by expanding the evidence base.
- Further theoretical work is needed to understand how diverse sources such as patient narratives and real-world data inform care delivery and research priorities.

## Background

For palliative care researchers, knowledge generated outside traditional academic institutions or dissemination mechanisms can help contextualise their work. This includes information produced by governments, industry, and organisations whose primary function is not publication, resulting in relevant materials such as reports, data, and guidelines, collectively known as ‘grey literature’.^1,2^

Grey literature comes in many forms (Table 1) and, unlike formally peer-reviewed research, is often decentralised, dispersed, and difficult to locate. This research, which may have been conducted by clinicians or healthcare administrators who lack the time and resources to publish their work in scholarly journals, can offer important insights into clinical practice, service delivery, and consumer experiences. This includes patient and carer perspectives captured through Patient-Reported Outcome Measures (PROMs) and values-based research.^1,3^ While peer-reviewed literature is more easily accessible through sophisticated search tools such as bibliographic databases, comprehensive retrieval of grey literature remains challenging and impractical for researchers. Yet manuals on systematic review methodology promote the inclusion of grey literature to increase the quality and depth of reviews and the strength of their conclusions.^4^

**Table 1.** Types of grey literature.

• Blogs • Conference papers, posters, and proceedings • Contact with primary researchers • Country profiles • Curriculum/course materials • Data • Diaries • Dissertations, theses • Fact sheets • Government documents • Guidelines • Handbooks • Images • Interviews	• Lectures • Legal documents • Legislation • Magazines • Manuals • Newsletters • Off-prints • Orations and speeches • Pamphlets and brochures • Podcasts • Policy documents • Preprints • Trade journals • Issue, discussion, and green and white papers • Press releases	• Questionnaires, surveys, and tests • Registers (including those for research studies) • Reports (technical and government) • Social media platforms • Software • Standards • Statistics • Videos • Webinars • Webpages and websites • Working documents and papers • Yearbooks

Recognising the significance of grey literature is important not only for those conducting systematic reviews with meta-analyses but also for researchers more broadly. An early study by Cook (2001) evaluated the contribution of grey literature searching to a palliative care systematic review but concluded that the effort yielded little return. However, this finding was based on limited methods, namely a single grey literature database (SIGLE), personal contacts, and a general call for information.^5^ Since then, tools and search engines have improved markedly, making it more feasible to identify and include high-quality grey literature. More recent commentary suggests that ‘[the benefits of including gray literature may far outweigh the cost in time and resources needed to search for it’, and that ‘a carefully thought out gray-literature search strategy . . . may be . . . invaluable’.^1^ Including grey literature can help minimise publication bias – the disproportionate publication of studies reporting positive results. It can also provide relevant information not captured in peer-reviewed publications.^1^

The volume and accessibility of grey literature has expanded significantly with digital technologies, enabling governments, NGOs, and research centres to publish policy-relevant materials more easily and at scale.^6^ This has repositioned grey literature as a prolific and influential evidence source. It is evident in the emergence of ‘living’ guidelines and systematic reviews which rely on ongoing evidence surveillance and offer benefits of improved currency, reduced research waste, and more timely knowledge translation.^7,8^ According to the Australian Living Evidence Collaboration, the living evidence approach broadens ‘the range of research and health-related data that can be included, such as real-world data from clinical quality registries and individual patient-level data’.^9^ In this vein, grey literature sources may therefore offer researchers a ‘more complete view of [the] available evidence’.^10^

Although the palliative care evidence base has expanded considerably since 2002,^11^ finding relevant evidence remains challenging. Unlike many clinical disciplines, palliative care extends beyond questions of clinical effectiveness to encompass quality of life, communication, equity, and systems of care. As such, it draws on a wide variety of research designs and perspectives, integrating empirical, practice-based, and policy-oriented evidence involving patients, carers, families, and care providers across health and social care sectors. Palliative care delivery is inherently interdisciplinary and often fragmented across primary, secondary, and tertiary services, requiring evidence that addresses continuity, coordination, and models of care.^12^ Furthermore, most peer-reviewed research originates from high-income countries and focusses primarily on cancer, leaving notable gaps in relation to other life-limiting conditions, diverse populations, and culturally responsive care.^11^ Palliative care research also receives relatively limited funding, even within the broader field of cancer research.^13^

In this context, grey literature plays a valuable complementary role. It can supplement the published evidence base by capturing real-world experiences, practice innovations, and service-level insights that are often underrepresented in academic literature. Given the field’s emphasis on access, equity, and person-centred care, grey literature may help illuminate systemic challenges and emerging responses, thereby informing practice, shaping policy, and broadening the research agenda.

Techniques and processes for locating grey literature have been catalogued in various fields, from digital health, school breakfast programmes, to asylum seekers.^14–16^ However, to our knowledge, no such process has been documented for the diffuse and multidisciplinary field of palliative care.

## Aim

The aim of this article is to present a framework to support researchers in systematically locating grey literature relevant to palliative care across broad range of sources. Practical guidance is also offered for reporting grey literature searches with the level of transparency required in systematic review or guideline publication.

## Methods

The palliative care grey literature framework was developed pragmatically in response to observed challenges in identifying relevant, high-quality grey literature in the field. Its design was informed by the authors’ combined experience as medical information specialists supporting systematic reviews and as palliative care academics supervising higher degree research students who often needed to seek evidence outside academic literature. Framework development followed an expert consensus approach within the authorship group. This drew on well-established principles for grey literature information used by health librarians (typically informally published online in the form of ‘Libguides’)^17,18^ and systematic review methodological guidelines such as those of JBI^19^ and the Cochrane Collaboration.^4^

First, broad approaches to locating palliative care research were clarified and documented. These approaches constitute the framework elements. Next, specific sources of grey literature were identified with potential relevance to palliative care research areas. Each source was systematically assessed for this relevance across palliative care and its many domains, stakeholders (including patients, families, and providers), and settings. Palliative care-specific sources of grey literature were automatically included while more general grey sources were tested for the depth, breadth, and accessibility of their palliative care content. This was determined by conducting a search in each using the following textwords: palliative, end-of-life, hospice, life-limiting, bereavement, terminal care, terminally ill.

## Results

**Figure 1.**
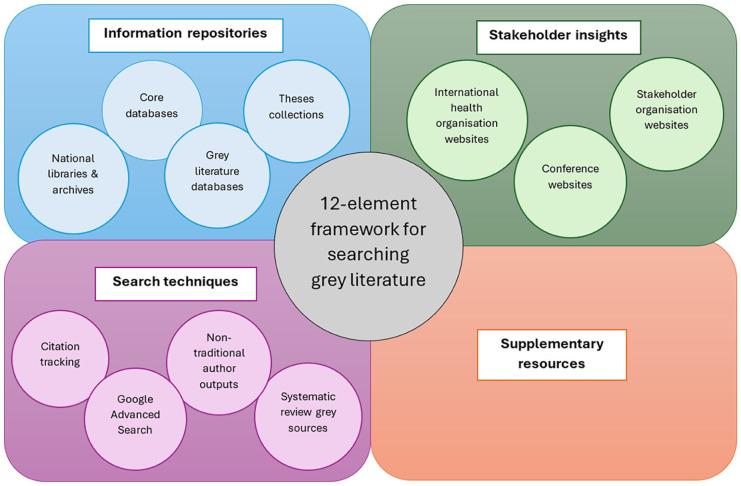
Framework for identifying grey literature in palliative care research.

Twelve categories of grey literature were identified and are presented here as a 12-element framework (Figure 1). To support clarity, the 12 elements have been grouped into four thematic zones: Information repositories, Stakeholder insights, Search techniques, and Supplementary resources. This thematic organisation reflects how researchers typically engage with different types of grey literature and supports adaptable use of the framework across diverse research contexts.

The elements are not intended to be routinely and comprehensively included in every palliative care grey literature search, regardless of topic or resources. Rather, they offer a structured and transparent approach to conceptualising and executing searches across a broad range of resource types. Researchers are encouraged to review the elements and associated sources as a team during the planning stage, adapting their use according to the specific research question, context, and available resources.

Table 2 lists recommended palliative-care relevant sources within each element. A checklist to support structured grey literature searching is provided in Supplemental Material S1.

**Table 2.** Key sources of palliative care grey literature by thematic zone.

Thematic zone	Category	Source suggestions
Information repositories	Core databases	• ACM Digital Library (technology focus) • African Index Medicus (AIM) • AgeLine • Index Medicus for the Eastern Mediterranean Region (IMEMR) • Index Medicus for the South-East Asia Region (IMSEAR) • Informit Health Collection (Australian focus) • IEEE Xplore Digital Library (technology focus) • Latin-American and Caribbean Center on Health Sciences Information (LILACS): Literature in Spanish, Portuguese, and English • PAIS Index (ProQuest): For public and social policy grey literature • WPRIM (Western Pacific Region Index Medicus)
	Grey literature databases	• BASE (Bielefeld Academic Search Engine) • Bookshelf (National Library of Medicine, US) • Digital Commons Network • ECRI Institute (US): A repository of grey literature on cost-effectiveness, safety, and quality of care across care settings worldwide. Section on palliative care evidence • Grey Matters (CADTH) • Health Systems Evidence (McMaster University, McMaster Health Forum) • Mednar: Federated medical deep web search engine • National Academies Press • OpenDOAR: Directory of Open Access Repositories • OpenGrey: Closed since 2021 • OpenMD: Documents from US government agencies, global health organisations, medical journals, and reference sites • National Grey Literature Collection (UK): No longer updated **Mixed content sources** • Canadian Institutes of Health Research (CIHR) • CORE: World’s largest collection of open access research papers taken from University repositories • OAIster: Digital resources from open archive collections includes theses, tech reports, research papers, image collections • ScienceOPEN.com • Social Science Research Network (SSRN): Medical preprints, conference papers, research papers, grey literature
	National libraries & archives	• British Library (UK) • Library and Archives Canada • Library of Congress (US) • National Library of New Zealand • Trove (National Library of Australia)
	Theses collections	• Theses Canada • EBSCO Open Dissertations • EThOS (British Library, UK) • Individual University Institutional open-access repositories • NDLTD Global ETD Search • OATD (Open Access Theses & Dissertations) • PHDData (Worldwide thesis database) • ProQuest Dissertations & Theses Global (PQDT Global) • Shodhganga (India, via INFLIBNET) • Trove (National Library of Australia)
Stakeholder insights	Stakeholder organisation websites	• African Palliative Care Association (APCA) • American Academy of Hospice and Palliative Medicine (AAHPM) • Asia Pacific Hospice Palliative Care Network • Australia New Zealand Society of Palliative Medicine (ANZSPM) • Canadian Hospice Palliative Care Association (CHPCA) • Center to Advance Palliative Care (CAPC) • European Association for Palliative Care (EAPC) • Hospice UK
		• International Association for Hospice and Palliative Care (IAHPC) • International Children’s Palliative Care Network (ICPCN) • Latin American Association for Palliative Care (ALCP) • Marie Curie (UK) • Multinational Organization of Supportive Care in Cancer (MASCC) • National Hospice and Palliative Care Organization (NHPCO) • Paediatric Palliative Care • PalCHASE (Palliative Care in Humanitarian Aid Situations and Emergencies) • Palliative Care Australia (PCA) • Palliative Care Nurses Australia (PCNA) • Palliative Care Research Society (UK) • Pallium Canada • Public Health Palliative Care International (PHPCI) • Worldwide Hospice Palliative Care Alliance (WHPCA)
	Conference websites	• American Academy of Hospice and Palliative Medicine (AAHPM) Annual Assembly • Asia Pacific Hospice Palliative Care Conference • Centre for Palliative Care Research and Education Conference (Australia) • Clinical Oncology Society of Australia Annual Scientific Meeting • European Association for Palliative Care (EAPC) World Congress • African Palliative Care Association (ACPA) Conference • International Palliative Care Network (IPCN) Conference • McGill International Congress on Palliative Care (Canada) • Marie Curie Research into Practice Conference (UK) • Oceanic Palliative Care Conference (OPCC) • Public Health Palliative Care International Conference (PHPCI) • International Dementia Conference • Scottish Partnership for Palliative Care (SPPC) Annual Conference • World Research Congress of the EAPC
	International health organisation websites	• PAHO IRIS (Pan American Health Organization) • World Health Organization – IRIS database
Search techniques	Citation tracking	• PubMed • Scopus • Web of Science
	Non-traditional author outputs	• The Conversation • Academic profile pages • Blogs • Social media mentions • Professional networking sites (e.g. LinkedIn)
	Grey sources in systematic reviews	• Agency for Healthcare Research and Quality (AHRQ) • CareSearch Systematic Review Collection • Cochrane Database of Systematic Reviews • Database of Promoting Health Effectiveness Reviews (DoPHER; EPPI Centre) • Epistemonikos • Health Evidence (McMaster University, Canada) • JBI • Open Science Framework (OSF) • Prospero (Register of clinically relevant systematic review protocols) • TRIP Pro
Supplementary resources	Additional selected resources	**Data sharing resources:** • Healthdata.gov • Our World in Data • Research Data Australia • UK Data Service **Trial registries:** • ClinicalTrials.gov • ICTRP Search Portal • Australian New Zealand Clinical Trials Registry
		**Practice guidelines:** • bpacNZ better medicine (NZ) • bcguidelines.ca (British Columbia guidelines) • NICE Evidence Search (UK): Archived since 2022 • GIN: International Guidelines Library • Practice based evidence in nutrition (PEN) **Policy resources:** • APO: Analysis and Policy Observatory (Australia) • Kings Fund (UK): Research and analysis on health and social care • Ministry of Health (NZ) • ELDIS (Institute of Development Studies) • European Observatory on Health Systems and Policies • OECD iLibrary • Overton: Cited as the world’s largest policy and grey literature database • Policy Commons **Law and legal:** • AustLII (Australian law and legislation) • Capital Monitor (LexisNexis) • End of Life Law for Clinicians: ELLC **Statistics:** • Australian Bureau of Statistics (ABS) • Australian Institute of Health and Welfare (AIHW) • OECD Health Statistics **Standards:** • Standards Australia • Australian Commission on Safety and Quality in Health Care (ACSQHC) • Aged Care Quality and Safety Commission (ACQSC)

## Framework elements

The 12 elements of the framework are described here.

### Information repositories

#### Core databases

Databases such as Embase, Scopus, and PsycEXTRA index conference abstracts and theses, offering access to emerging or unpublished work.

#### Grey literature databases

Databases like Health Systems Evidence, BASE, WHO IRIS, and aggregators like Overton and OpenMD offer structured access to policy reports and institutional outputs. Discovery tools like OpenDOAR and Mednar can guide users to source sites.

#### National libraries and archives

Resources like Trove, the British Library, and Library and Archives Canada offer access to grey literature including reports, theses, and historical documents, often with national relevance.

#### Theses collections

Open-access repositories such as Trove, EThOS, OATD, and NDLTD house postgraduate research, which is often rich in methods or underreported findings.

### Stakeholder insights

#### Stakeholder organisation websites

Websites of professional bodies and agencies such as Hospice UK or the American Academy of Hospice and Palliative Medicine host reports, white papers, and evaluations that may not be captured by search engines.

#### Conference websites

Some journals publish abstracts and even posters from select conferences in supplements, for example those from the European Association for Palliative Care World Congress are included in *Palliative Medicine*. This can make them findable in databases (Step 2). However, not all conference abstracts are formally published in this way. Conference websites can be, therefore, a rich source of emerging research in the form of abstracts, posters, and presentations.

#### International health organisation websites

World Health Organization (WHO), and the Pan American Health Organization provide access to reports, data, and policy frameworks relevant to global palliative care.

### Search techniques

#### Citation tracking

**Figure 2.**
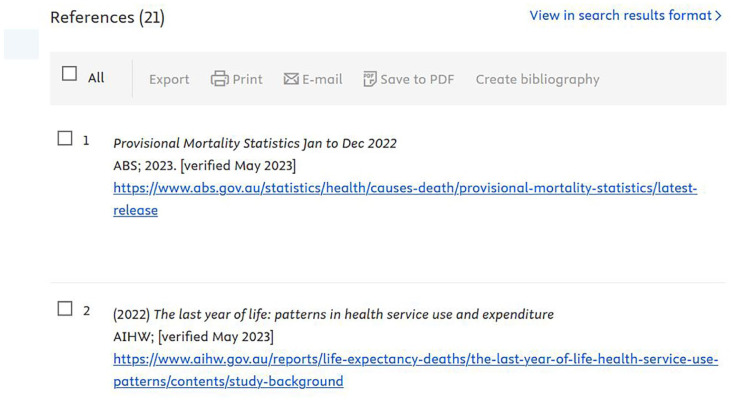
Grey literature sources in the reference list of an article in Scopus.

Citation tracking can uncover relationships between studies to identify further potentially relevant papers. This may be conducted backwards or forwards.^20^ Backwards tracking involves starting with a paper deemed relevant to the topic and checking its reference list for other relevant papers. Some databases also facilitate this process – particularly PubMed, Scopus, and Web of Science. In Scopus, grey literature is shown in the article’s reference list, often with a URL to the full document (Figure 2).

#### Grey sources in existing systematic reviews

Reviewing methods sections of relevant systematic reviews can reveal databases, repositories, and websites used by other researchers to identify grey sources. Key sources of systematic reviews include Cochrane, JBI, CareSearch Systematic Review Collection, and Epistemonikos. Reference lists may also yield grey literature not indexed in traditional databases.

#### Google Advanced search

Google Advanced Search can help identify grey literature when used with Boolean logic, domain, or file-type limits (e.g. site:.gov.au and filetype:pdf), and private browsing to reduce bias.^16^

- Teams should predefine screening limits (e.g. 10 pages or 100 links) based on the research team’s time and available resources. Note the limit to the number of results screened along with the rationale in reporting.^21^

#### Non-traditional authors outputs

Outputs such as blogs, podcasts, and media articles may be located via ORCID, LinkedIn, ResearchGate, or X (formerly Twitter). The Conversation provides credible, accessible commentary.

### Supplementary resources

#### Additional, selected resources

Additional sources include data-sharing platforms, clinical trial registries, professional guidelines, legal resources, and statistical agencies. These provide authoritative sector-specific content.

### Documenting the search process

Cochrane advises transparency in grey literature search documentation, so that searches can be ‘reproducible to the extent that this is possible’.^4^ PRISMA 2020 and PRISMA-Search, an extension of the PRISMA statement for reporting literature searches in reviews, suggest that, at a minimum, researchers should report:

1. The types of grey literature targeted (e.g. theses, reports, and policy documents).
2. The strategies used (e.g. citation tracking, Google Advanced Search, and repository searches).
3. Resources accessed and search dates.^21,22^

For example:

A Google Advanced Search was conducted in incognito mode on 19 March 2025 using the terms “good death” AND “voluntary assisted dying” OR “VAD” OR “physician assisted dying.” Searches were limited to Australian domains (.gov.au; .edu.au) and file type PDFs. The first 100 results per search were screened.

Researchers may include a table of websites and repositories searched accompanied by search dates as an appendix. Unlike database results, grey literature is not expected to appear in PRISMA flow diagrams. Rather, only the grey literature sources assessed for inclusion in the review should be documented. Researchers are encouraged to consult the PRISMA-S extension for reporting literature searches in systematic and scoping reviews.^21^

## Discussion

Searching for grey literature poses known challenges, even for experienced researchers. Teams should define in advance how deeply to search and where to look.^23^ In palliative care, these challenges may be amplified due to its multidisciplinary, multi-setting approach, which includes families as well as patients, and encompasses physical, psychosocial, and spiritual dimensions.^24^ Although developed through expert consensus by palliative care researchers and health information specialists, the 12-element framework may also be generalisable to other health disciplines. While specific sources will differ, the approach offers a flexible starting point for developing templates to support grey literature searching.

In developing the framework, several practical issues emerged. Firstly, many sources offer limited search functionality, often restricted to basic keyword searching. This can increase the time and effort required. Determining when to stop searching can also be difficult. Predefining limits based on a restricted number or types of sources, reviewer workload, or expected yield may help. One study applied the concept of ‘data saturation’ from qualitative research, ceasing the search when no additional information was found offering new insights.^25^

Grey literature often yields large, varied datasets. Items may lack abstracts or clear titles, complicating documentation and relevance assessment. Managing this data is typically manual, requiring spreadsheets rather than reference software and careful documentation to support screening decisions.

Although the framework separates sources by type, inevitably some overlap and duplication will occur across the elements. While this mirrors the duplication that happens when searching multiple databases, identifying and removing duplicates in grey literature searches is a manual task, adding further workload.

Despite impeccable record-keeping, grey literature searches often prove difficult to reproduce. Web-based resources can be transient, with URLs changing, sources disappearing completely, or ceasing to be updated. Even well-established grey repositories such as DART-Europe E-Theses Portal and OpenGrey, can become outdated or decommissioned.

Appraising grey literature is also challenging. Unlike journal articles, many grey literature sources lack clear authorship, methodological detail, or peer review, making it difficult to assess their rigour or trustworthiness. Quality may be variable, and biases can arise from selective dissemination, advocacy-driven content, or unclear provenance. Tools such as the AACODS checklist offer a structured way to consider authority, accuracy, coverage, objectivity, date, and significance, but judgement is still required.^26^ There is little agreement about what constitutes quality in grey literature, particularly when the purpose of the literature may be descriptive, contextual, or persuasive rather than evaluative. In addition to practical barriers, conceptual tensions remain around how different forms of knowledge such as lived experience narratives, community-generated data, and real-world service evaluations are valued and integrated into evidence syntheses.^27^ Further theoretical development may be needed to understand how such diverse sources contribute to models of care and healthcare delivery.^28,29^

A discussion on improving grey literature searches for palliative care knowledge must acknowledge both the growing role of social media in its dissemination, and the potential for generative artificial intelligence (GenAI) technologies to enhance retrieval. Social media platforms such as LinkedIn, X, and ResearchGate have enhanced research visibility and facilitated connections among scholars.^30^ Moreover, they increase the speed at which non-commercial publications, data, and other information is disseminated, enabling timelier access to relevant, current information. While this can enhance access to high-quality grey literature for researchers and clinicians, it also risks spreading unverified, low-quality, or erroneous information, requiring caution.

Regarding GenAI, the authors take a cautious stance. These technologies can automate literature searches, reducing time and costs, improving reproducibility, and helping locate obscure materials in a way previously unavailable.^31^ However, there is a need to differentiate between those resources built on trusted academic intelligence like SCOPUS AI and others like ChatGPT which are large language models built on a vast range of publicly available data, and may generate unreliable or fabricated responses.^32^ Despite the corporate hyperbole and industry claims that GenAI can serve as a ‘trusted guide[s] through the vast expanse of human knowledge’,^33^ concerns about ethics, safety, accuracy, technological limitations, and transparency suggest the need for ongoing human oversight, critical appraisal, and a (healthy) scepticism regarding its capacity to deliver purported benefits in the short- to medium-term.

### Limitations

Several limitations are noted. First, while a broad array of grey literature sources relevant to palliative care has been identified, others may exist. Periodic updates to the framework are recommended to account for new sources and emerging technologies. Second, the framework is largely restricted to English-language resources, which may limit its global applicability. Furthermore, although the framework is grounded in methodological expertise and experiential validation, formal empirical evaluation is lacking. Testing via a systematic review case study approach, as used in public health by Adams et al.,^25^ or a Delphi process to tailor the framework and associated sources to palliative care research priorities, would be valuable next steps.

## Conclusion

This paper presents a 12-element framework to support a systematic, adaptable approach to identifying and reporting grey literature in palliative care research. It is intended to assist researchers in incorporating diverse sources of evidence, enhancing the relevance, inclusivity, and applicability of findings in palliative care practice.

## Supplemental Material

sj-docx-1-pmj-10.1177_02692163251381487 – Supplemental material for Beyond black and white: A framework for identifying grey literature in palliative care research Supplemental material, sj-docx-1-pmj-10.1177_02692163251381487 for Beyond black and white: A framework for identifying grey literature in palliative care research by Raechel Damarell, Seth Nicholls, Jessica Tyndall and Caroline Phelan in Palliative Medicine
