# Supplementary material for: Beyond black and white: A framework for identifying grey literature in palliative care research
Source: Palliat Med. 2025 Oct 23;39(10):1109–18. doi: 10.1177/02692163251381487 (PMC12640360; doi:10.1177/02692163251381487)
Supplement: sj-docx-1-pmj-10.1177_02692163251381487 – Supplemental material for Beyond black and white: A framework for identifying grey literature in palliative care research [file sj-docx-1-pmj-10.1177_02692163251381487.docx]

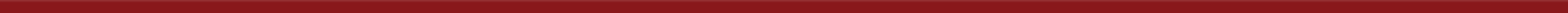


**Systematically searching for grey literature: A checklist for palliative care researchers**

Systematic reviews

Examples: Cochrane Database of Systematic Reviews; Joanna Briggs Institute Systematic Reviews; Campbell Collaboration; Prospero database; AHRQ Evidence Reports; Other options

Core databases

Examples: CINAHL; Embase; Emcare; PsycInfo; PubMed/Medline; Scopus; UpToDate; Web of Science; Other options

**National libraries & archives**

Examples: Trove (National Library of Australia); WorldCat; Library and Archives Canada; British Library; Other options

**Stakeholder organisations**

Examples: International Association for Hospice & Palliative Care; Hospice UK; CareSearch Palliative Care Knowledge Network; Palliative Care Australia; Other options

Conference websites

Examples: Oceanic Palliative Care Conference; Asia Pacific Hospice Palliative Care Conference; Centre for Palliative Care Research and Education (CPCRE); Other options

Health organisations

Examples: World Health Organization - IRIS Database OECD iLibrary; Other options

Theses collections

Examples: Open Access Theses and Dissertations; EBSCO Open Dissertations; DART-Europe E-theses Portal; Networked Digital Library of Theses and Dissertations: Global ETD search; Other options

Citation tracking

Examples: Scanned relevant reference lists; Scopus; PubMed; Other options

Advanced Google Search

Searched Google using Advanced Mode;

Used Anonymous mode; Limited to file type PDFs; Varied site or domain options (e.g., .edu or .ac.uk)

**Grey literature databases**

Examples: Mednar; PsycExtra; CareSearch Grey Literature Database; Other options

Tracking key authors

Examples: The Conversation; Academic profile pages; LinkedIn; blogs; Other options

Additional resources

OpenDOAR; Registry of Open Access Repositories; Social Sciences Research Network; Mendeley Data; Research Data Australia; ICTRP Search Portal; clinicalTrials.gov; GIN: International Guidelines Library; UK Data Service

Damarell R, Nicholls S, Tyndall J, Phelan C. Beyond black and white … *Palliative Medicine*. 2025. doi:[10.1177/02692163251381487](https://doi.org/10.1177/02692163251381487)
